# A multicenter, prospective, randomized, open-label, blinded endpoint trial of intravenous thrombolysis with tenecteplase for acute non-large vessel occlusion in extended time window (OPTION): Rationale and design

**DOI:** 10.1515/jtim-2025-0048

**Published:** 2025-10-25

**Authors:** Gaoting Ma, Ran Mo, Yingting Zuo, Shujuan Meng, Yifan Wu, Ziying Jiang, Jieying Wu, Shaoyuan Lei, Thanh N. Nguyen, Jeffery L. Saver, Duolao Wang, Lianmei Zhong, Qingfeng Ma, Junwei Hao

**Affiliations:** Department of Neurology, Xuanwu Hospital Capital Medical University, National Center for Neurological Disorders, Beijing, China; Beijing Municipal Geriatric Medical Research Center, Beijing, China; Key Laboratory for Neurodegenerative Diseases of Ministry of Education, Beijing, China; Department of Evidence-Based Medicine, Xuanwu Hospital Capital Medical University, Beijing, China; Department of Neurology, Radiology, Boston Medical Center, Chobanian & Avedisian School of Medicine, Boston University, Boston, MA, USA; Department of Neurology, Geffen School of Medicine at UCLA, UCLA Comprehensive Stroke Center, UCLA, Los Angeles, CA, USA; Global Health Trials Unit, Liverpool School of Tropical Medicine, Liverpool L3 5QA, United Kingdom

**Keywords:** acute ischemic stroke, intravenous thrombolysis, tenecteplase, randomized trial, protocol

## Abstract

**Background and Objectives:**

Recombinant human tenecteplase (TNK) tissue-type plasminogen activator (rhTNK-tPA, also named tenecteplase) is non-inferior to alteplase when given up to 4.5 hours after acute ischemic stroke (AIS) within 4.5 hours. Whether intravenous tenecteplase compared with supportive care improves the outcome of patients with AIS due to non-large vessel occlusion (non-LVO) between 4.5-24 hours is uncertain. The Tenecteplase for Acute Non-large vessel occlusion in the Extended Time Window (OPTION) trial aims to test the efficacy and safety of intravenous tenecteplase in patients presenting between 4.5-24 hours after symptom onset who have a non-LVO and evidence of salvageable tissue on perfusion imaging.

**Methods:**

OPTION is a multicenter, prospective, randomized, open-label, blinded endpoint (PROBE) study. Patients with AIS due to non-LVO and evidence of salvageable brain tissue within 4.5-24 hour time window meeting eligibility criteria will be allocated in a 1∶1 ratio to tenecteplase or standard medical treatment. A target mismatch profile is defined as ischemic core volume <50 mL, a mismatch ratio ≥1.2, and a mismatch volume ≥10 mL. A total of 568 patients will provide >80% power to detect a 12% absolute difference in the proportion of patients achieving an excellent functional outcome (modified Rankin scale [mRS] 0-1 at 90 days) at the two-sided 0.05 significance level. The primary efficacy outcome is excellent functional outcome (mRS 0-1) at 90 days. Secondary efficacy outcomes include disability level (ordinal distribution of mRS) at 90 days, functional independence (mRS 0-2) at 90 days, reperfusion at 24 hours, infarct volume at 24 hours, early clinical response at 24 hours, the National Institutes of Health Stroke Scale (NIHSS) change from baseline at 7 days/discharge, health status and quality of life at 90 days. Safety outcomes include symptomatic intracranial hemorrhage within 36 hours, systemic bleeding within 90 days, and mortality within 90 days.

**Discussion:**

The OPTION trial (NCT05752916) will provide evidence regarding the efficacy and safety of tenecteplase 4.5 to 24 hours in patients with AIS due to non-LVO.

## Introduction

Intravenous thrombolysis (IVT) is recommended for acute ischemic stroke (AIS) in eligible patients up to 4.5 h from symptom onset.^[1]^ However, only a small proportion of AIS patients receive IVT, mainly due to the narrow therapeutic time window.^[2]^ An individual patient-level meta-analysis of 8 trials involving IVT without mechanical thrombectomy (MT) for AIS beyond 4.5 h in patients predominantly showing imaging evidence of salvageable tissue demonstrated superior 90-day functional outcomes compared with standard medical care, despite a higher incidence of symptomatic intracranial hemorrhage (sICH).^[3]^ These findings provide evidence-based data to support further randomized controlled trials (RCTs) evaluating IVT in extended time windows for AIS patients with varying vascular occlusion sites.

Five recent RCTs assessed the possibility of extending the IVT time window to 24 h after symptom onset in patients with ischemic stroke.^[4, 5, 6, 7, 8]^ The tenecteplase reperfusion therapy in acute ischemic cerebrovascular events III (TRACE-III) trial demonstrated that in patients with confirmed anterior circulation large vessel occlusion (LVO), extended-window tenecteplase without access to MT achieved superior functional outcomes compared with standard medical care.^[4]^ However, the thrombolysis in imaging-eligible, late window patients to assess the efficacy and safety of tenecteplase (TIMELESS) trial, which also used tenecteplase in the extended time window for patients with anterior circulation LVO, most of whom underwent subsequent MT, did not show improved functional outcomes.^[5]^ Recently, the treatment with intravenous alteplase in ischemic stroke patients with onset time between 4.5 and 24 hours (HOPE) trial evaluated IVT with alteplase in patients with salvageable brain tissue within 4.5 to 24 h, showing functional benefit.^[6]^ The Chinese Acute Tissue-Based Imaging Selection for Lysis in Stroke-Tenecteplase II (CHABLIS-T II) trial revealed that tenecteplase increased reperfusion without symptomatic intracranial hemorrhage in patients selected by imaging in late-time window treatment.^[7]^ The Extending the Time Window for Thrombolysis in Posterior Circulation Stroke without Early computed tomography (CT) Signs (EXPECTS) trial demonstrated that in patients with predominantly mild posterior circulation stroke who did not undergo mechanical thrombectomy (MT), IVT administered 4.5 to 24 h after onset led to superior functional outcomes compared with standard medical care.^[8]^

Despite the progress so far, the efficacy and safety of IVT administered beyond 4.5 h after symptom onset in AIS patients with non-large vessel occlusion (non-LVO) remains inadequately studied. Based on vessel size, large arteries include the internal carotid artery, M1 segment of the middle cerebral artery (MCA), vertebral arteries, and basilar artery.^[9]^ Relative to LVO, AIS presentations related to non-LVO are due to medium vessel occlusion, small vessel (deep and long pial penetrator) occlusion, hemodynamic watershed ischemia, and uncommon or disseminated conditions.^[9, 10, 11]^ The exact prevalence of non-LVOs remains incompletely characterized, as most epidemiological studies lack systematic intracranial vascular imaging protocols. Available data from population-based studies and large clinical registries indicate that approximately 54%-76% of AIS presentations are estimated to be attributable to non-LVO.^[12, 13, 14]^

Advanced imaging may help to select AIS patients who could benefit from IVT in the extended or unknown time window. These advanced imaging techniques include CT or magnetic resonance-based perfusion imaging, as well as magnetic resonance imaging (MRI) with diffusion-weighted imaging-fluid-attenuated inversion recovery (DWI-FLAIR) mismatch.^[15]^ To date, the efficacy and safety of magnetic resonance imaging (MRI)-based thrombolysis in wake-up stroke (WAKE-UP),^[16]^ extending the time for thrombolysis in emergency neurological deficits (EXTEND),^[17]^ TRACE-III,^[4]^ HOPE,^[6]^ and EXPECTS^[8]^ trials have demonstrated the efficacy of IVT beyond the 4.5-hour time window. Four of these trials incorporated advanced imaging selection criteria, while the EXPECTS trial did not mandate advanced imaging-based selection. Notably, 73 (31.2%) were enrolled solely on the basis of clinical presentation and noncontrast CT findings.^[8]^ Based on current evidence, advanced imaging in an extended or unknown time window is still considered as a requirement for providing IVT therapy.^[1,18]^

As the gold-standard thrombolytic agent for AIS since 1995, intravenous alteplase remains a first-line therapy for eligible patients.^[18]^ Recombinant human tenecteplase (TNK) tissue-type plasminogen activator (rhTNK-tPA, also named tenecteplase), a genetically modified variant of alteplase, exhibits higher fibrin specificity and longer half-life, allowing single bolus administration. Recently, at least five phase 3 RCTs demonstrated comparable efficacy and safety profiles of intravenous tenecteplase versus alteplase up to 4.5 h after stroke onset, prompting an update of stroke management guidelines to recommend tenecteplase as one of the preferred thrombolytic agents.^[19, 20, 21, 22, 23]^

We therefore designed the Tenecteplase for Acute Non-large Vessel Occlusion in Extended Time Window (OPTION) trial to investigate the efficacy and safety of intravenous tenecteplase in patients presenting between 4.5-24 h after symptom onset who have a non-LVO and evidence of salvageable tissue on perfusion imaging.

## Materials and methods

### Trial design

**Figure 1:**
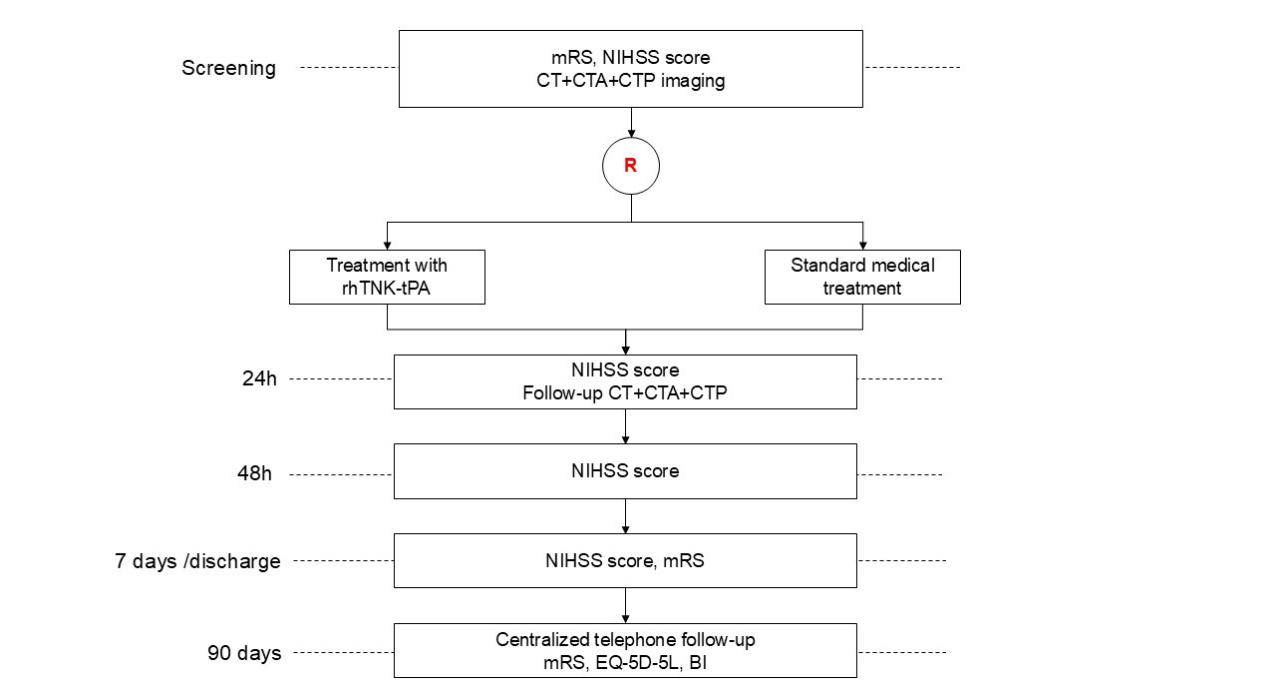
Study Flow Chart. mRS, modified Rankin scale; NIHSS, National Institutes of Health Stroke Scale; CT, computed tomography; CTA, computed tomography angiography; CTP, computed tomography perfusion imaging; rhTNK- tPA, recombinant human tenecteplase tissue-type plasminogen activator; EQ-5D-5L, EuroQoL 5-Dimensions 5-Level questionnaire; BI, Barthel Index.

OPTION is a phaseⅢ, multicenter, prospective, randomized, open-label, blinded endpoint (PROBE), controlled clinical trial, aiming to investigate the superiority of intravenous tenecteplase, compared with standard medical treatment, to increase 90-day excellent outcome in patients with acute non-LVO stroke and evidence of salvageable brain tissue on baseline computed tomography perfusion (CTP) imaging who are treatable between 4.5-24 h of symptom onset (Figure 1). The OPTION trial is being conducted in compliance with the Declaration of Helsinki principles and is registered at ClinicalTrials. gov (NCT05752916). The protocol has received approval from Institutional Review Boards (IRB) at all participating sites before enrollment (IRB with Xuanwu Hospital, Capital Medical University with number [2022]205). Written informed consent was obtained from all subjects or from their legally authorized representatives before the study. The trial is being performed in approximately 50 centers in China.

### Patient population

Patients with AIS due to non-LVO presenting between 4.5-24 h from last-seen-well (including wake-up stroke and unwitnessed stroke), baseline the National Institutes of Health Stroke Scale (NIHSS) from 6 to 25 (or 4 to 5 with disabling deficit) and target mismatch profile on CTP (defined as ischemic core volume <50 mL, a mismatch ratio ≥1.2, and a mismatch volume ≥10 mL) are enrolled into this trial. Based on these imaging criteria, the study population primarily includes patients with acute intracranial non-large vascular occlusions, including the M2 (vertical MCA branches) or more distal segments of MCA, anterior cerebral artery (ACA), posterior cerebral artery (PCA), anterior inferior cerebellar artery (AICA), posterior inferior cerebellar artery (PICA), superior cerebellar artery (SCA), as well as those with large- to medium-vessel stenosis, or a combination of both. The detailed inclusion and exclusion criteria are presented in Table 1.

**Table 1:** Inclusion and Exclusion Criteria

Inclusion criteria
Clinical inclusion criteria
□ Clinical diagnosis of acute ischemic stroke
□ Age ≥18 years
□ Pre-stroke mRS score 0-1
□ Disabling stroke, defined as follows:
Baseline NIHSS score 6-25 at the time of randomization, Or NIHSS 4-5 with disabling deficit (*e.g*. hemianopia, aphasia, loss of hand function) as determined by the managing clinician
□ Symptom onset within 4.5-24 h of treatment initiation (onset time refers to “last-seen-well”)
□ Written informed consent from patients or legally responsible representatives
Imaging inclusion criteria
□ Target mismatch profile on CTP defined as an ischemic core volume (using rCBF <30% on CTP) <50 mL, a mismatch ratio (Tmax >6 sec volume to ischemic core ratio) ≥1.2, and a mismatch volume (Tmax >6 sec volume- ischemic core volume) ≥10 mL
Exclusion criteria
Clinical exclusion criteria
□ Treatment with a thrombolytic within the last 72 h or intention to receive IVT
□ Contraindications to thrombolysis
□ Planned or anticipated treatment with EVT
□ Rapidly improving symptoms, particularly if in the judgement of the managing clinician is likely to result in a NIHSS <4 at randomization
□ Pregnancy or lactating; formal testing needed in woman of childbearing potential
□ Cerebral neoplasm (with mass effect)
□ Hereditary or acquired hemorrhagic diathesis, coagulation factor deficiency
□ Impairment in coagulation due to comorbid disease or anticoagulant use. If on warfarin, international normalized ratio (INR) >1.7 or prothrombin time >15 s; if use of any direct oral anticoagulant within the last 48 h; if use of heparin/heparinoid within the last 24 h
□ Use of glycoprotein Ⅱb-Ⅲ a inhibitors within the last 72 h
□ Baseline platelet count <100,000/μL
□ Undergoing hemodialysis or peritoneal dialysis; known severe renal insufficiency with glomerular filtration rate <30 mL/min or serum creatinine >220 mmol/L (2.5 mg/dL)
□ Suspected aortic dissection
□ Major surgery or biopsy within the last 1 month
□ Any active bleeding or recent bleeding within the last 1 month, e.g. gastrointestinal or urinary tract hemorrhage
□ Known severe, life-threatening allergy (more severe than skin rash) to contrast agents
□ Severe, uncontrolled hypertension (systolic blood pressure >185 mmHg or diastolic blood pressure >110 mmHg)
□ Any terminal illness such that the patient would not be expected to survive more than half a year
□ Current participation in any investigational study that may confound outcome assessment of the study
□ Any condition that, in the judgement of the investigator, is inappropriate for participation in the trial or could impose hazards to the patient (*e.g*. inability to understand and/or follow the study procedures and/or follow-up due to mental disorders, cognitive or emotional disorders)
Imaging exclusion criteria
□ Acute intracranial hemorrhage
□ Acute LVO on MR/CT angiography, including ICA, MCA-M1, vertebral artery and basilar artery

CT: computed tomography; CTP: computed tomography perfusion; EVT: endovascular thrombectomy; ICA: internal carotid artery; IVT: intravenous thrombolysis; LVO: large vessel occlusion; MCA: middle cerebral artery; MR: magnetic resonance; mRS: modified Rankin Scale; NIHSS: National Institutes of Health Stroke Scale; rCBF: regional cerebral blood flow; Tmax: time-to-maximum.

### Randomization and blinding

Patients are randomized, in a 1 : 1 ratio, using a minimization process to receive either intravenous tenecteplase or standard medical treatment, stratified by vessel occlusion location (anterior or posterior circulation), age (<65 or ≥65 years), baseline NIHSS (<16 or ≥16) and center. The randomization will be done by a computerized central interactive web response system. All endpoints will be evaluated by an independent investigator who is unaware of the treatment group.

### Intervention

Patients allocated in the interventional arm will receive intravenous tenecteplase at 0.25 mg/kg, not exceeding a maximum dose of 25 mg. Each vial of tenecteplase is reconstituted with 3 mL of sterile water for injection and injected based on body weight in a single bolus over 5-10 seconds.

Patients allocated in the control arm will receive antiplatelet therapy (aspirin or clopidogrel alone) at the discretion of local investigators.

All enrolled patients are treated according to the *2023 Chinese Guidelines for Diagnosis and Treatment of Acute Ischemic Stroke* and *2022 Chinese Guideline for the Secondary Prevention of Ischemic Stroke and Transient Ischemic Attack*.^[24,25]^ Patients who are planned or anticipated to be treated with endovascular thrombectomy (EVT) are excluded from the trial. No antiplatelet therapy (*e.g*. aspirin, clopidogrel, ticagrelor, or glycoprotein IIb/IIIa inhibitors) should be administered within 24 h after tenecteplase. Oral or parenteral anticoagulants (*e.g*. warfarin, dabigatran, heparin, low molecular weight heparin, argatroban) are prohibited for the first 24 h in both arms. Close attention should be paid to patient’s changes of symptoms and signs in order to detect bleeding events in time. The management of bleeding events should follow the clinical routine and be taken immediately.

### Clinical and imaging evaluation

Study visits are performed at the screening period, 0 h, 24 (-2/+12) hours, 48 (±6) hours, 7 (±2) days/discharge (whichever is earlier), and 90 (±7) days. During the screening period, all participants undergo CT, computed tomography angiography (CTA), and CTP after clinical evaluation. Clinical examination and radiological follow-up with CT, CTA, and CTP are performed at 24 (-2/+12) hours. If symptom deterioration with suspected intracranial hemorrhage occurs during hospitalization, an immediate CT scan should be performed. A non-contrast CT is recommended to be done, if possible, at 7 (±2) days or at discharge. At baseline and follow-up visits, clinical evaluation includes vital signs, concomitant medications, NIHSS and modified Rankin Scale (mRS) score. At 90 (±7) days, mRS, EuroQoL 5-Dimensions 5-Level questionnaire (EQ-5D-5L) and Barthel Index (BI) are assessed *via* a standardized telephone interview. All trial procedures are summarized in Table 2.

**Table 2:** Trial Schedule

Procedure/Investigation	Screening	Treatment	Follow-up			
	V0	V1	V2	V3	V4	V5
Time	Baseline	0 h	24 (-2/+12) h	48 (±6) h	7 (±2) days/discharge^g^	90 (±7) days
Informed consent	X					
Inclusion/exclusion criteria	X					
Demographic data^a^	X					
Medical history^b^	X					
Concomitant medication	X		X	X	X	X
Vital signs^c^	X		X	X	X	
ECG	X					
Laboratory tests^d^	X		X			
Imaging^e^	X		X		X^*^	
mRS^f^	X				X	X
NIHSS	X		X	X	X	
GCS	X		X	X	X	
EQ-5D-5L						X
BI						X
Randomization	X					
Study medication administration		X				
AEs/SAEs		X	X	X	X	X
Study completion						X

AEs: Adverse Events; BI: Barthel index; ECG: electrocardiography; EQ-5D-5L: EuroQoL 5-Dimensions 5-Level questionnaire; GCS: Glasgow Coma Scale; mRS: Modified Rankin Scale; NIHSS: National Institutes of Health Stroke Scale; SAEs: Serious Adverse Events. ^a^: Demographic data include date of birth, sex, ethnic group, body height, body weight. ^b^: Medical history includes history of present illness, past medical history, family history, drug allergies, smoking and alcohol use. ^c^: Vital signs include temperature, blood pressure and heart rate. ^d^: Laboratory tests include hematology, liver and renal function, coagulation profile and glucose; pregnancy test is limited to women of child bearing potential. ^e^: Brain CT+CTA+CTP. ^f^: Baseline mRS is defined as a pre-stroke score. ^g^: Whichever is earlier. *: Repeat CT is recommended, if possible; if symptom deterioration with suspected intracranial hemorrhage occurs during hospitalization, an immediate CT scan should be performed; X: The assessment required at each time point.

If a suspected or confirmed serious adverse event (SAE) occurs, necessary assessments should be performed according to the protocol. The SAEs will be reviewed by the Clinical Events Committee (CEC).

### Primary outcome

The primary outcome is the proportion of excellent functional outcome defined as an mRS of 0-1 at 90 (±7) days.

### Secondary outcomes

There are seven secondary outcomes, (1): Disability level (ordinal mRS score) at 90 (±7) days; (2): Proportion of functional independence (mRS of 0-2) at 90 (±7) days; (3): Proportion of reperfusion at 24 (-2/+12) h, defined as >90% reduction in Tmax >6 s lesion volume by CTP; (4): Infarct volume at 24 (-2/+12) h, measured by CT scan at 24 (-2/+12) h; (5): Proportion of early clinical response at 24 (-2/+12) h, defined as a NIHSS score of 0 or 1 or NIHSS drop of ≥8 from baseline; (6): NIHSS change from baseline at 7 (±2) days/discharge (whichever is earlier); (7): Health status and quality of life (EQ-5D-5L) at 90 (±7) days.

### Safety outcomes

The safety outcomes are (1): Proportion of patients with sICH within 36 h, as defined by Heidelberg bleeding classification;^[26]^ (2): Proportion of systemic bleeding within 90 (±7) days, as defined by The Global Utilization of Streptokinase and Tissue Plasminogen Activator for Occluded Coronary Arteries (GUSTO): moderate and severe bleeding; (3): Mortality within 90 (±7) days.

### Sample size estimation

The sample size is based on the primary outcome. Based on the reported pooled data of the stroke thrombolysis trials in the extended time window,^[27]^ we expect a 12% absolute difference in the proportion of patients reaching the primary outcome between patients treated with tenecteplase and standard medical treatment (expected rate of excellent functional outcome 50% in patients treated with tenecteplase and 38% in patients treated with standard medical treatment). Considering the potential impact of one interim efficacy analysis on the probability of type I error, we adjusted the alpha error to a two-sided alpha of 0.049. The total sample size of 568 (*n* = 284 per treatment group) will have 80% power to detect the specified difference at the two-sided 0.049 significance level, with a 5% dropout rate.

One efficacy interim analysis is planned when 50% of the target sample size has completed 90-day follow-up. A Lan-DeMets alpha spending function will be used to control the overall alpha level, using O’Brien and Fleming boundaries (corresponding to an alpha level of 0.003 and 0.049 at the interim and final analysis, respectively).

### Statistical analysis

The analysis population for the efficacy analyses will be performed on the intention-to-treat (ITT) population. For the primary outcome, the proportions of mRS 0-1 at 90 (±7) days will be compared between two treatment groups using a modified Poisson regression. The risk ratio (RR) and its 95% confidence interval (CI) will be reported. We will also perform adjusted analysis of the primary outcome with adjustment for pre-specified covariates.

The distribution of mRS score at 90 (±7) days will be analyzed using ordinal logistic regression, with treatment effect reported as a common odds ratio and its corresponding 95% CI. If the proportional odds hypothesis is rejected, the generalized odds ratio (GenOR) with 95% CI will be calculated. For binary secondary efficacy endpoints, modified Poisson regression model will be employed to determine RR and its 95% CI. For continuous secondary efficacy endpoints, linear regression models will be employed to estimate mean difference as a measurement of treatment effect. In case the normality assumption for the residuals is violated, win ratio will be calculated. Safety analyses will be performed on the safety population who received one of the study interventions according to the treatment they actually received. Safety endpoints will be compared using the modified Poisson regression. Subgroup analyses include age (<65 years and ≥ 65 years), time from symptom onset (last-seen-well) to randomization (>4.5-9 h and >9-24 h and stroke on awakening), baseline NIHSS score (<10 and ≥10), vessel occlusion location (anterior circulation and posterior circulation), occlusion site (M2 segment of MCA, M3-M4 segment of MCA, ACA, PCA and stenosis), baseline serum glucose (<100 mg/dL and ≥100 mg/dL) and baseline systolic blood pressure (≤140 mmHg and >140 mmHg).

Three safety interim analyses were planned when the first 100, 200, 300 patients have completed the 90-day follow-up. One efficacy interim analysis was planned to be conducted when 50% of the total patients have completed the 90-day follow-up. All statistical analyses will be performed following a statistical analysis plan (SAP) which will be finalised before the database lock and unblinding of the trial. SAS software version 9.4 (SAS Institute) and R version 4.4.1 will be used for the statistical analyses.

### Data safety and monitoring board

An independent data safety and monitoring board (DSMB) will regularly monitor the safety of the trial and guarantee the safety of all patients. The DSMB will perform safety reviews when the first 100, 200 and 300 patients are enrolled and complete 90-day follow-up. If there are concerns about safety, the DSMB will make a recommendation to the trial steering committee about continuing, stopping or modifying the trial. One prespecified efficacy interim analysis will be performed when 284 patients have completed 90-day follow-up. Results of the interim analysis will only be presented to the DSMB.

Due to the fast speed of enrollment, after review with the DSMB in June 2025, the original interim analysis plan was considered no longer to serve its purpose. On the recommendation of the DSMB, the steering committee rescinded the planned efficacy interim analysis and the third safety analysis. Since no formal assessment of efficacy is done and no alpha is spent, we will not adjust the alpha level or p values for the final analysis.

### Study organization and funding

OPTION is an investigator initiated clinical trial. It is sponsored by Yangfan 3.0 Diagnosis and Treatment Capability Enhancement Project (ZLRK202514) and CSPC Recomgen Pharmaceutical (Guangzhou) Co., LTD (Guangzhou, China). The funders have no role in the design, conduct, statistical analysis or reporting of the results. A steering committee provides scientific and strategic guidance for the trial. An executive committee manages the day-to-day activities of the trial. Endpoint events, safety endpoints and SAEs are reviewed by a CEC and all clinical imaging data are independently reviewed by an imaging core laboratory.

## Discussion

The OPTION trial is a pivotal study designed to evaluate the efficacy and safety of intravenous tenecteplase in patients with AIS presenting within 4.5-24 h from symptom onset, who have a non-LVO and demonstrate salvageable tissue on perfusion imaging. However, unlike the conspicuous identification of LVO, accurate localization of the occlusion site in non-LVO often poses a diagnostic challenge. We utilize a pragmatic imaging selection approach, enrolling patients who demonstrate salvageable penumbral tissue after exclusion of LVO. The advantages of this imaging selection are that it can exclude patients with clear indications for MT, and also enhance detection rates of distal and posterior circulation vessel occlusions by combining CTA with CTP.^[28,29]^ Another ongoing randomized trial evaluating tenecteplase in patients with non-LVO treated in the extended time window is Randomization to Extend Stroke Intravenous ThromboLysis In Evolving Non-Large Vessel Occlusion With TNK (RESILIENT EXTEND-IV, NCT05199662). The databases will be pooled after both trials are completed and published.

## Conclusions

The OPTION trial will provide evidence regarding the efficacy and safety of tenecteplase 4.5 to 24 h in patients with AIS due to non-LVO.
